# LPI Radar Waveform Recognition Based on Hierarchical Classification Approach and Maximum Likelihood Estimation

**DOI:** 10.3390/e26110915

**Published:** 2024-10-28

**Authors:** Kiwon Rhee, Jaeyoung Baik, Changhoon Song, Hyun-Chool Shin

**Affiliations:** 1 Department of Intelligent Semiconductors, Soongsil University, Seoul 06978, Republic of Korea; kwrhee@ssu.ac.kr; 2 Department of Information and Communication, Soongsil University, Seoul 06978, Republic of Korea; bjynvh@gmail.com; 3 EW AI & Jamming Technology R&D, LIG Nex1 Co., Ltd., Suwon 16347, Republic of Korea; changhoon.song@lignex1.com

**Keywords:** radar, waveform recognition, low probability of intercept

## Abstract

The importance of information gathering is emphasized to minimize casualties and economic losses in warfare. Through electronic warfare, which utilizes electromagnetic waves, it is possible to discern the enemy’s intentions and respond accordingly, thereby leading the battle advantageously. Consequently, related research is actively underway. The development of various radar signal modulation techniques has revealed limitations in the existing modulation recognition methods, necessitating the development of distinguishing features to overcome these limitations. This paper proposes and analyzes distinguishing features that can differentiate various modulation schemes. Eleven distinguishing features were employed, and twenty-two types of modulated signals, including analog, digital, and composite modulation, were classified using hierarchical classification approach and maximum likelihood estimation (MLE). The proposed method achieves a recognition performance of 99.76% at an SNR of 20 dB and 98.45% at an SNR of 8 dB.

## 1. Introduction

Since the advent of the mass production of weapons due to the development of industrial technology, warfare has predominantly been consumptive in nature. However, in modern times, the trend has shifted toward quickly ending wars with strategies based on rapid and accurate information, thereby minimizing damage. Consequently, the importance of electronic warfare has emerged, as it enables the identification of enemy intentions based on collected information and the pre-emptive development of countermeasures. This allows for the neutralization of enemy command, control, and communications at the onset of hostilities, thereby establishing dominance in warfare.

Electronic warfare refers to all military applications utilizing electromagnetic waves and is broadly categorized into electronic attack, electronic protection, and electronic warfare support. Among these, electronic warfare support involves activities aimed at detecting, intercepting, and identifying electromagnetic waves emitted by the enemy to locate signals and their sources. By analyzing the enemy’s electromagnetic waves and utilizing the obtained information, efficient strategies and countermeasures can be prepared in advance, making electronic warfare support capabilities a crucial factor in determining the outcome of a war [1,2].

In particular, the technology for identifying enemy radar signals is critical as it allows for understanding the purpose of radar usage and the level of threat, thereby enabling the preparation of appropriate countermeasures in advance. However, with advancements in technology, communication signals are being covertly embedded within radar pulses, signal strength is being reduced, various pulse compression techniques and modulation methods are diversifying, and the number of operational radars is increasing, leading to a more complex signal environment on the battlefield [3,4,5]. In such a complex electromagnetic environment, radar signal recognition technology with high recognition rates and strong robustness is essential.

Traditional radar signal recognition used basic identification factors such as carrier frequency, time of arrival, pulse width, and pulse amplitude. However, in the increasingly complex signal environment of modern battlefields, these traditional identification factors are insufficient for distinguishing between electromagnetic waves with similar variables. To address this issue, research has been conducted based on information about various modulation methods and machine learning [6]. In [7], 18 types of radar waveforms were differentiated using visual graph-based features along with k-nearest neighbor (kNN) and support vector machine (SVM) methods. In [8], eight types of single-modulation signals were classified using Wigner–Ville distribution (WVD)-based features and a binary tree approach. Ref. [9] identified five types of single-modulation signals based on pseudo-Zernike moments and kNNs. In [10], IPCA and SVMs were used to distinguish eight types of radar waveform.

In recent years, deep learning [11] has made remarkable progress and has begun to be applied in the field of radar modulation signal recognition research. For instance, Ref. [12] classified signals using binarized time–frequency images and deep residual learning. Ref. [13] generated time–frequency images based on smooth pseudo-Wigner–Ville distribution and used a DCNN model to classify eight types of radar waveforms. Ref. [14] utilized a CNN to extract features and employed a DQN-based classification network. Ref. [15] used an FF-CNN to extract features and distinguish seven types of single/composite-modulation signals. Ref. [16] extracted features with BiLSTM to differentiate six types of PSK signals. According to [17], the classification of 10 types of radar waveforms was performed using BLCDAE and X-net.

Deep-learning-based methods often show higher recognition accuracy compared to traditional machine learning methods. However, they require a large amount of pre-existing data and impose higher demands in terms of storage and computation due to the need for high-quality time–frequency images. These high requirements pose challenges for practical field applications. Therefore, it is crucial to evaluate whether traditional machine learning methods can still exhibit a competitive performance in radar signal recognition.

We propose a modulation recognition method that sequentially distinguishes various single-modulation signals and composite-modulation signals from broad categories to individual signal classifications. This method utilizes a hierarchical classification, time–frequency spectrogram-based features obtained through short-time Fourier transform (STFT), and maximum likelihood estimation (MLE) [18] to find the distribution that maximizes the likelihood of the observed features.

The remainder of this paper is structured as follows. In Section 2, we introduce the proposed method, including time–frequency transformation, feature extraction of modulation signals, hierarchical classification, and MLE. In Section 3, we present the experimental results. Finally, in Section 4, we draw conclusions based on the experimental results.

## 2. Proposed Methods

**Figure 1:**
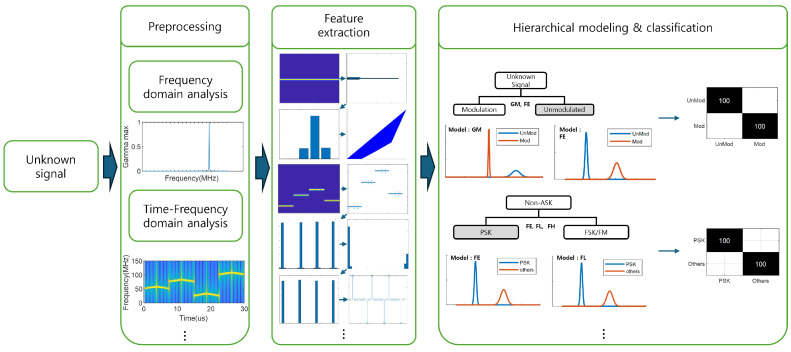
The flow chart of the proposed method.

In Section 2, we present a radar signal recognition method based on preprocessing, feature extraction, and a hierarchical classification with maximum likelihood estimation. To analyze radar signals, we used short-time Fourier transform (STFT) to generate time–frequency spectrograms of the radar signals. Subsequently, we extracted 11 features based on the I and Q signals and the time–frequency spectrogram. Finally, to perform signal recognition, we designed a hierarchical classification model with maximum likelihood estimation model that selectively uses the 11 extracted features. The overall flow of the proposed method is illustrated in Figure 1.

### 2.1. Time–Frequency Transformation

To obtain the time–frequency information of radar signals, we used short-time Fourier transform (STFT). For more accurate time–frequency information, research has explored applying Wigner–Ville distribution (WVD) [5] or Choi–Williams distribution (CWD) [5]. However, the computational load of WVD is approximately 32 times higher than that of STFT [19], and the computational load of CWD is about 3 times higher than that of WVD [20]. Therefore, these methods are known to have excessively high computational demands, making them impractical for real-world applications.

Given the observed radar signal x(τ), the short-time Fourier transform (STFT) is defined as follows:

(1) $$X\left(k,t\right)=\int_{-\inf}^{\inf}x\left(\tau \right)\omega \left(\tau -t\right)e^{-j2\pi k\tau }d\tau$$

Here, *t* and *k* represent the time and frequency indices, respectively, and ω(τ−t) denotes the window at time *t*. We extracted features through the analysis of the radar signal in both the frequency domain and the time–frequency domain.

### 2.2. Feature Extraction

In this section, we define both traditional features and new features to distinguish various single- and composite-modulation signals.

#### 2.2.1. Gamma Max (GM)

**Figure 2:**
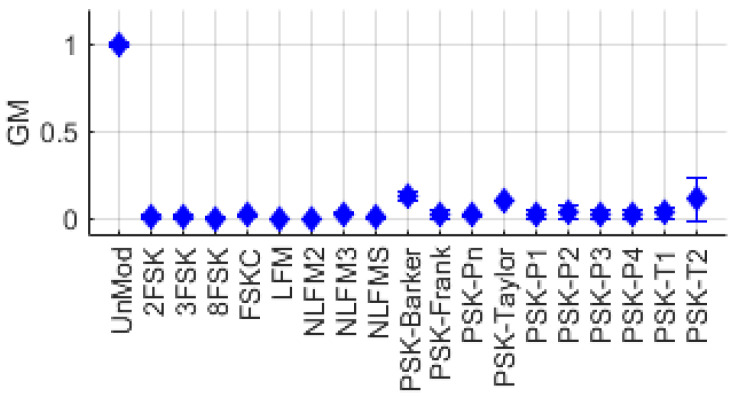
Gamma max distribution for various single-modulation signals.

A representative feature using the power spectrum, GM, represents the maximum power of the spectrum [21]. When the collected signal x(τ) is normalized, GM is observed to be highest in unmodulated signals (UnMod) while it is relatively lower in other modulated signals. Figure 2 shows the distribution of GM extracted from UnMod signals, as well as FSK, FM, and PSK series single-modulation signals. UnMod signals have values close to 1, whereas other signals show values closer to 0.

(2) $$GM=\frac{1}{N}\max_{n}\frac{1}{N}\left|\sum_{\tau =0}^{N-1}x\left(\tau \right)e^{-j2\pi n\tau /N}\right|$$

#### 2.2.2. Gini’s Coefficient (GC)

GC is a feature that expresses the unevenness of multiple discrete values [22]. We obtained the signal D(k) by summing the time-axis information of the time-frequency spectrogram X(k,t) over all time instances *t*

(3) $$D\left(k\right)=\sum_{t=1}^{T}X\left(k,t\right)\;$$

The signal D(k) will have a bandwidth where the signal is present, and the regions outside this bandwidth will observe only AWGN noise power. We assumed the minimum value in D(k) as the AWGN noise component and removed it using a difference operation.

(4) $$D^{\prime }\left(k\right)=D\left(k\right)-\min_{k}D\left(k\right)\;$$

**Figure 3:**
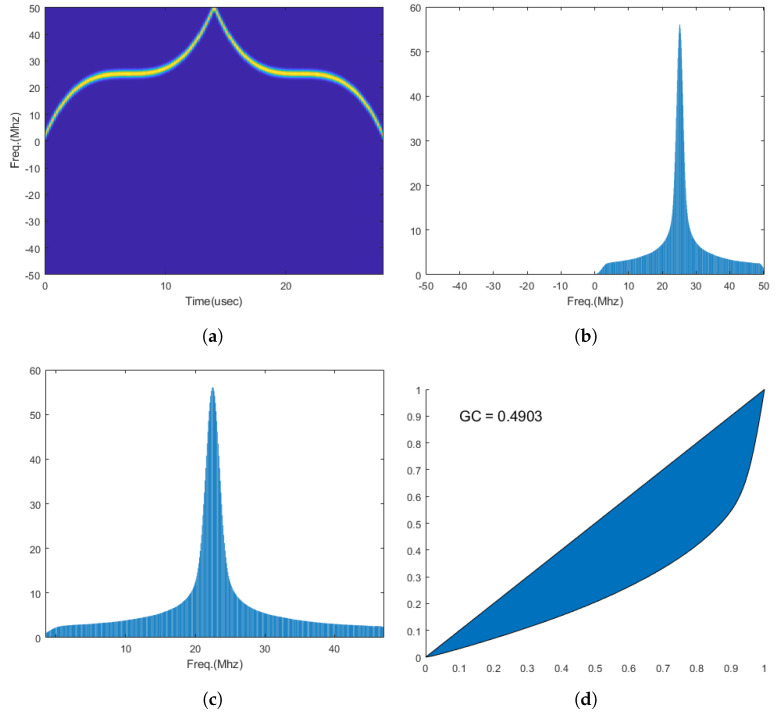
GC acquisition process. (**a**) Spectrogram of NLFM3, (**b**) accumulated sum to the frequency axis of (**a**), (**c**) a valid signal band of (**b**), (**d**) Lorenz curve of (**c**).

**Figure 4:**
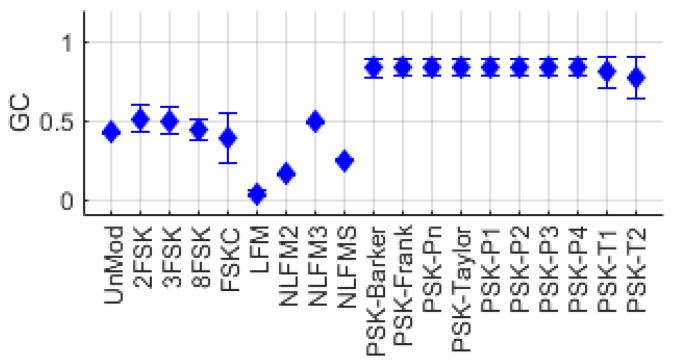
Gini’s coefficient distribution for various single-modulation signals.

Subsequently, to determine the bandwidth occupied by the signal, we designated the 5th and 95th percentile of the cumulative sum of $D^{\prime }\left(k\right)$ as the start and end points $i^{\prime }$ and $i^{\prime \prime }$, respectively. We then calculated the GC using the values of $D^{\prime }\left(k\right)$ between $i^{\prime }$ and $i^{\prime \prime }$. Figure 3 illustrates the process of calculating GC. As shown in Figure 4, the GC observed in PSK series modulation signals has generally higher values compared to the GC observed in FM/FSK series modulation signals.

#### 2.2.3. Frequency Entropy (FE)

Information entropy is a metric that quantifies the uncertainty of a signal [23]. We quantified the probability of frequency occurrence using information entropy. Based on $D^{\prime }\left(k\right)$ from Equation (4), the probability of frequency occurrence is calculated as follows:

(5) $$p_{s}\left(k\right)=\frac{D^{\prime }\left(k\right)}{\sum_{k}D^{\prime }\left(k\right)}\;$$

FE can be calculated using $p_{s}\left(k\right)$ as follows:

(6) $$FE=-\sum_{k=i^{\prime }}^{i^{\prime \prime }}p_{s}\left(k\right)\log p_{s}\left(k\right),\;$$

**Figure 5:**
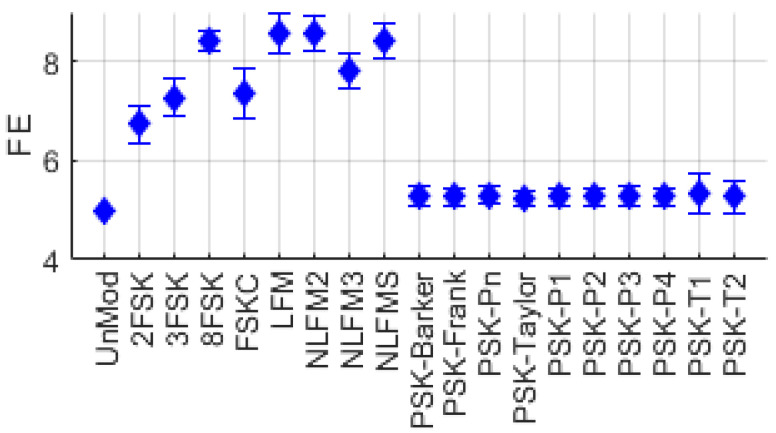
Frequency entropy distribution for various single-modulation signals.

Figure 5 shows the distribution of FE for UnMod, FSK, FM, and PSK series single-modulation signals. It can be observed that the distributions of UnMod and PSK series signals are distinctly separated from those of FSK and FM series signals.

#### 2.2.4. Frequency Level (FL)

One of the features that distinguishes modulation signals is the number of frequency shifts. To detect the number of frequency shifts, we estimated the instantaneous frequency by using the index of the maximum value observed at each moment in the spectrogram S(k,t).

(7) $$\sum =\left[S_{1},S_{2},\cdots ,S_{T}\right]=\left[\begin{array}{ccc} S_{1,1} & \cdots & S_{1,T}\\ \vdots & \ddots & \vdots \\ S_{K,1} & \cdots & S_{K,T} \end{array}\right]$$

(8) $$I_{k}=F_{\hat{k_{t}}},\hat{k_{t}}=arg\max_{k}S_{t},t=1,2,\cdots ,T$$

**Figure 6:**
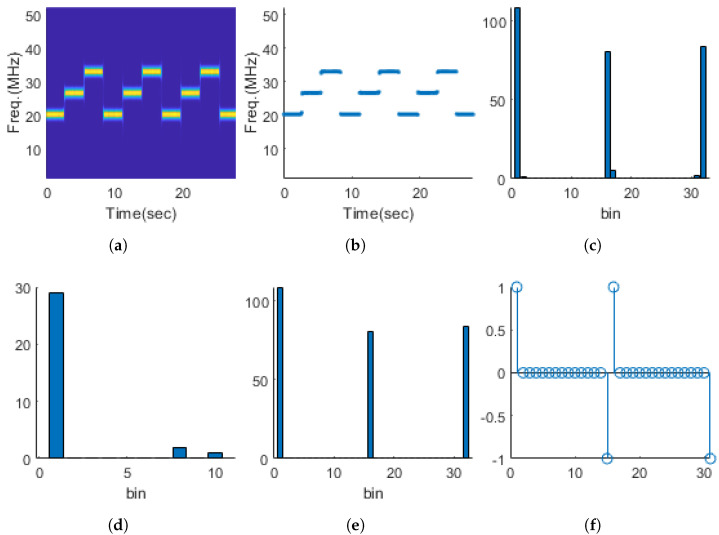
FL acquisition process. (**a**) Spectrogram of ternary FSK, (**b**) instantaneous frequency of (**a**), (**c**) histogram of (**b**), (**d**) histogram of (**c**), (**e**) significant frequency components extracted using (**d**), (**f**) difference operation of (**e**).

In Equations (7) and (8), *F* is the frequency axis, *k* is the frequency index, *t* is the time index, and *K* and *T* are the maximum values of the frequency and time indices, respectively. Next, we used a median filter to remove distortions in the instantaneous frequency caused by noise or transition processes. The third step is to divide the instantaneous frequency bandwidth into 32 segments and compute the first histogram. The first histogram obtained in this way may still contain noise or frequency components other than the instantaneous frequency. To remove these components, the first histogram is divided into 10 segments to compute the second histogram. In the second histogram, if we assign a frequency occurrence count of 0 in the first histogram for frequency ranges below the first observed empty space, only significant frequency components will remain in the first histogram. Subsequently, we define FL as the number of intervals with a value of 0 plus 1, obtained through a difference operation. The process of calculating FL is illustrated in Figure 6.

**Figure 7:**
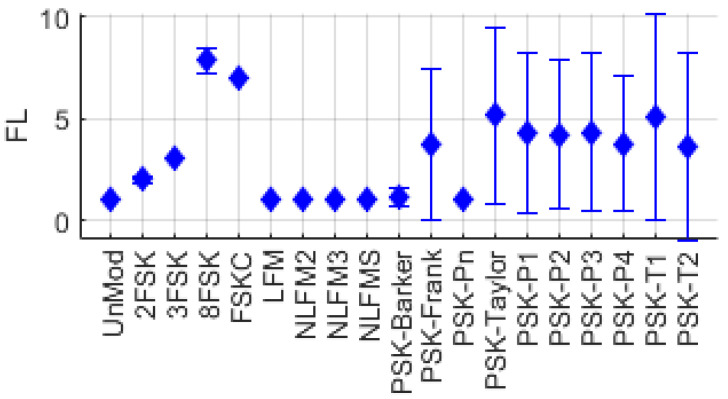
Frequency-level distribution for various single-modulation signals.

Figure 7 shows the distribution of FL for each single-modulation signal. It can be observed that FL has a value of 1 for UnMod and FM series signals, while, for FSK series signals, FL corresponds to the number of frequency tones in the signal.

#### 2.2.5. Frequency Hopping (FH)

**Figure 8:**
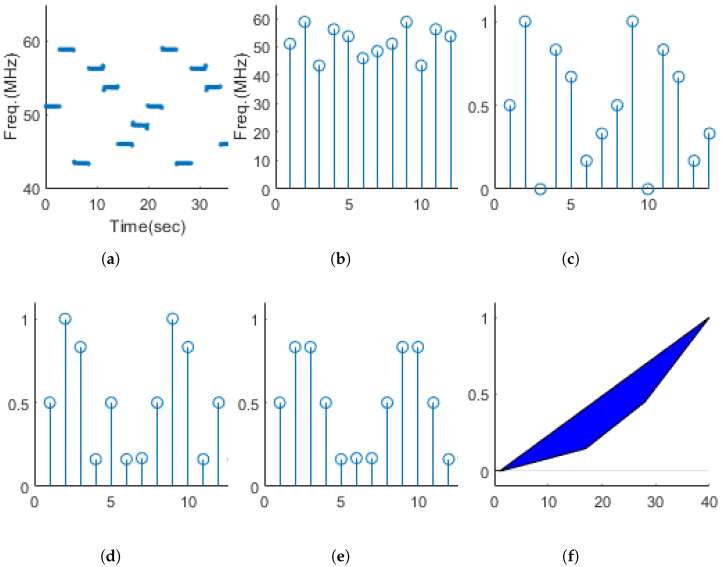
FH acquisition process. (**a**) Instantaneous frequency of FSK Costas, (**b**) symbols of (**a**), (**c**) normalization of (**b**), (**d**) difference operation of (**c**), (**e**) 3-sample median filtering for (**d**), (**f**) Lorenz curve of (**e**).

The random change in frequency is called frequency hopping. To determine the presence of frequency hopping, we performed the process illustrated in Figure 8. The first step is to estimate the instantaneous frequency from the spectrogram. In the second step, the median value between transition points is extracted as the symbol value. In the third step, the extracted symbol values are normalized to a range between 0 and 1. In the fourth step, the result from the third step is subjected to a difference operation. In the case of continuously incoming pulses, a large difference value appears at the points where the pulses change. To remove these, the fifth step involves performing 3-sample median filtering. The symbol values after performing the fifth step are used to calculate Gini’s coefficient, which is defined as FH. Since frequency hopping does not occur in UnMod and FM signals, only one symbol exists within one cycle of signal. In such cases, Gini’s coefficient cannot be calculated, so we assign a value of −1 to indicate this exceptional situation.

**Figure 9:**
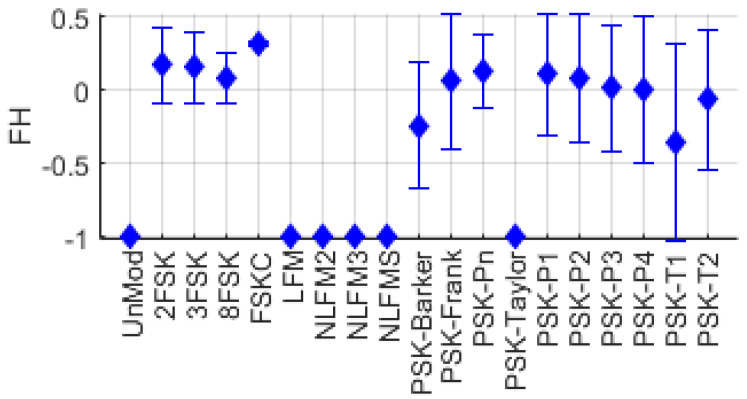
Frequency hopping distribution for various single−modulation signals.

The distribution of FH for single-modulation signals is shown in Figure 9. It can be observed that FSK series signals, which have frequency hopping, generally have high values, whereas UnMod and FM series signals have very low values.

#### 2.2.6. Inter-Pulse Entropy (ITE)/Inter-Pulse Gini’s Coefficient (ITG)

**Figure 10:**
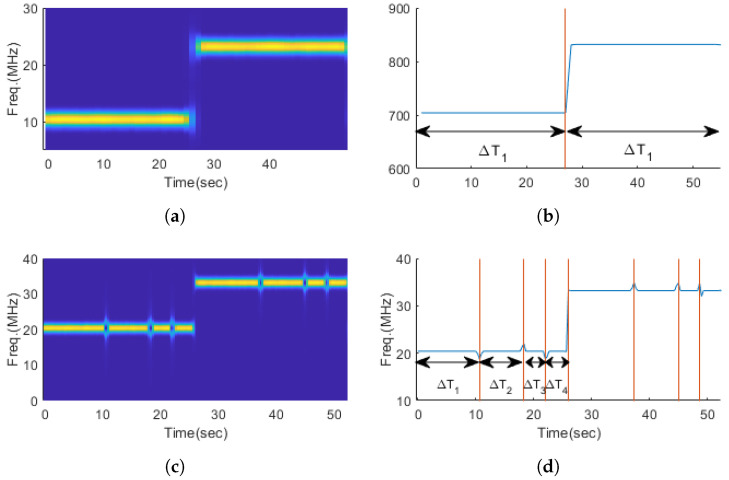
Comparison of the length of the phase maintenance section of FSK2, FSK2-PSK signals. (**a**) Spectrogram of FSK2 signal, (**b**) instantaneous frequency and phase maintenance interval of FSK2 signal, (**c**) spectrogram of FSK2-PSK signal, (**d**) instantaneous frequency and phase maintenance interval of FSK2-PSK signal.

To determine whether PSK signals are used as an internal modulation scheme within FSK or FM signals, we extracted the length of the intervals where the phase does not change within a symbol or pulse and calculated the information entropy and Gini’s coefficient. Figure 10 shows an example of extracting the length of the intervals where the phase does not change within a symbol for 2FSK modulation signals and 2FSK-PSK composite-modulation signals. For 2FSK single-modulation signals, the length of the intervals where the phase does not change within a symbol is equal to the length of the symbol. However, for composite-modulation signals, the lengths of these intervals within a symbol vary. Let the set of times during which the phase remains constant be $\Delta T=\{\Delta T_{1},\Delta T_{2},\Delta T_{3},...\}$. The information entropy and Gini’s coefficient calculated using ΔT are defined as ITE and ITG, respectively.

**Figure 11:**
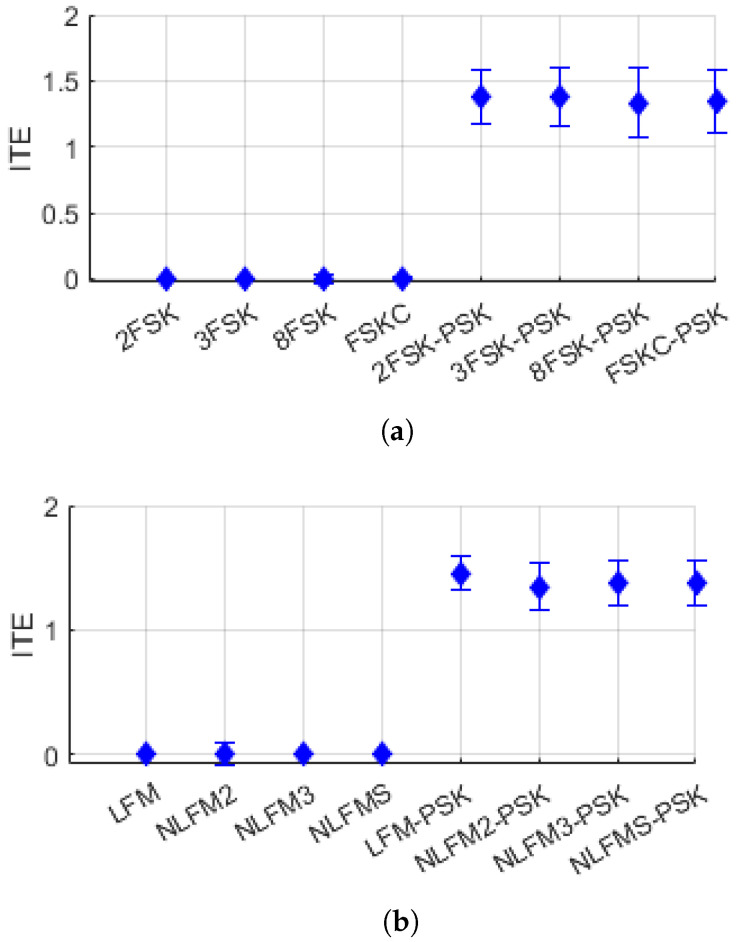
Distribution of ITE: (**a**) FSK and FSK-PSK signal, (**b**) FM and FM-PSK signal.

**Figure 12:**
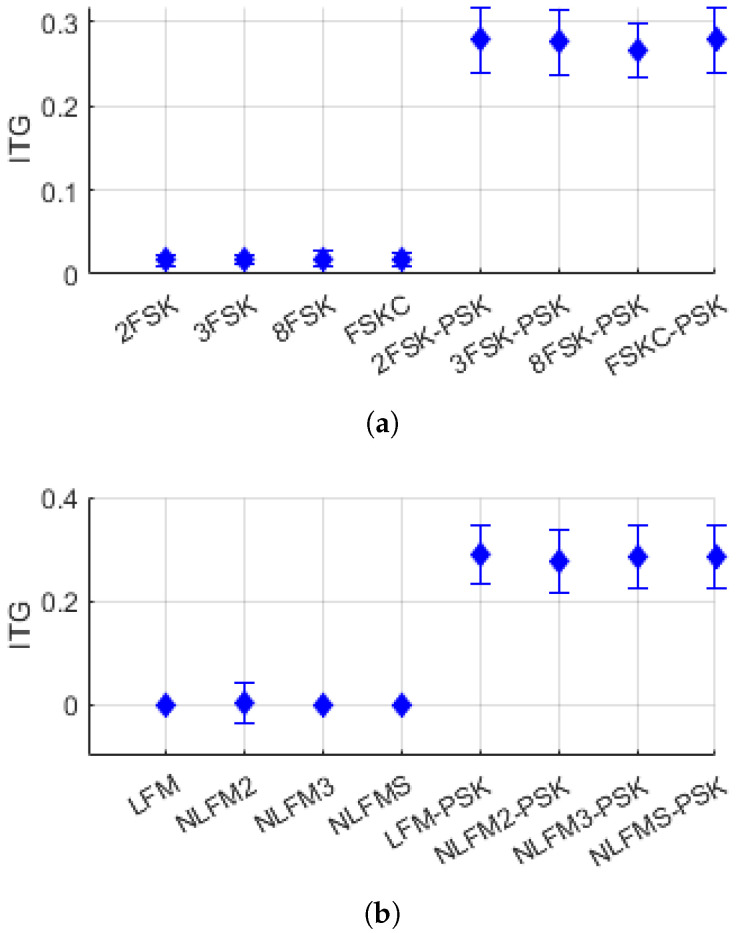
Distribution of ITG: (**a**) FSK and FSK-PSK signal, (**b**) FM and FM-PSK signal.

Figure 11 and Figure 12 show the distribution of the features ITE and ITG for FSK/FM single-modulation signals and FSK-PSK/FM-PSK composite-modulation signals. In both figures, it can be observed that the ITE and ITG values are low for single-modulation signals and relatively high for composite-modulation signals.

#### 2.2.7. Intra-Pulse Entropy (INE)/Intra-Pulse Gini’s Coefficient (ING)

INE and ING are features used to recognize changes in the instantaneous frequency within a symbol, such as upward or downward shifts, in FSK composite-modulation signals. We divide the symbols based on the phase duration times $\Delta T_{1},\Delta T_{2},\Delta T_{3},\ldots$ within the repetitive modulation signals. Then, the changes in the instantaneous frequency $D_{1},D_{2},D_{3},\ldots$ within a symbol are defined as a vector as follows:

(9) $$I_{k}=\left\{\begin{array}{c} D_{*}|\left(k-1\right)M+D_{min}<=D_{*}<kM+D_{min},\\ k=1,2,\cdots ,K-1\\ D_{*}|\left(k-1\right)M+D_{min}<=D_{*}<=kM+D_{min},\\ k=K \end{array}\right.$$

(10) $$M=\frac{D_{max}-D_{min}}{K}$$

In Equations (9) and (10), *D* represents the instantaneous frequency within a symbol, $D_{*}$ is the quantized instantaneous frequency of *D*, and *K* is the number of bins for quantization. In this study, *K* is set to 10. The ratio of the number of elements in the set $I_{k}$ to the total number of elements in *D* can be expressed as the probability $p_{k}$.

(11) $$p_{k}=\frac{I_{k}}{D}$$

**Figure 13:**
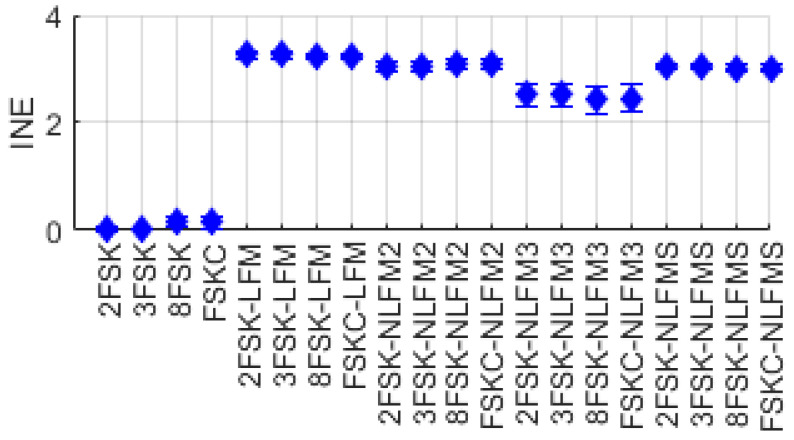
Distribution of INE for FSK and FSK-FM signal.

**Figure 14:**
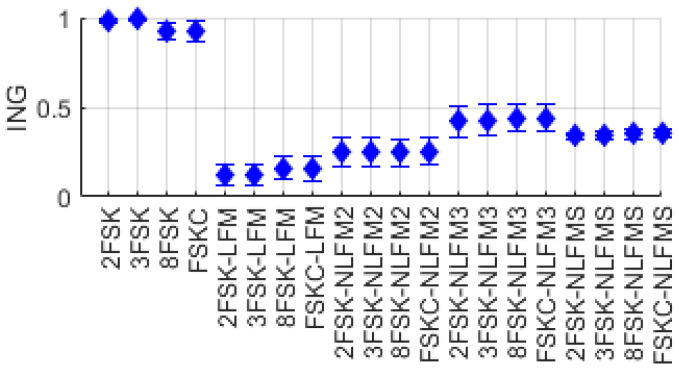
Distribution of ING for FSK and FSK-FM signal.

We calculated the information entropy and Gini’s coefficient based on the probability $p_{k}$. Let *L* be the number of symbols in one cycle of the signal. Then, we calculated the information entropy and Gini’s coefficient during each symbol duration, and INE and ING were averaged over one cycle of signals. Figure 13 and Figure 14 show the distribution of the features INE and ING for FSK single-modulation signals and FSK-FM composite-modulation signals. As shown in Figure 13, single-modulation signals with no changes in instantaneous frequency within a symbol have INE values close to 0, whereas composite-modulation signals exhibit relatively higher values. As shown in Figure 14, the feature parameter ING, unlike INE, has values close to 1 for FSK single-modulation signals, while exhibiting relatively lower values for FSK-FM composite-modulation signals.

#### 2.2.8. Nullity

When amplitude shift keying (ASK) is combined with other modulation signals, silent intervals occur within the overall signal duration, leading to the characteristic that the central frequency is not observed. We set a threshold to detect these silent intervals as the mean of the time–frequency spectrum power S(k,t), as shown in Equation (12).

(12) $$S_{th}=\frac{\sum_{k=1}^{K}\sum_{t=1}^{T}S\left(k,t\right)}{KT}$$

In Equation (12), *T* and *K* are the maximum indices of time and frequency, respectively. Then, based on the threshold value, the time–frequency spectrum power is binarized as shown in the equation.

(13) $$\begin{array}{c} \left[Y_{1},Y_{2},\ldots ,Y_{T}\right]=\left[\begin{array}{ccc} X_{1,1} & \cdots & X_{1,T}\\ \vdots & \ddots & \vdots \\ X_{K,1} & \cdots & X_{K,T} \end{array}\right],\\ X_{f,t}=\left\{\begin{array}{c} 1,S\left(k,t\right)>=S_{th}\\ 0,S\left(k,t\right)<S_{th} \end{array}\right. \end{array}$$

For each binarized vector $Y_{1},Y_{2},\ldots ,Y_{T}$, we calculated Gini’s coefficient. Let $g_{*}$ be the vector of Gini’s coefficients. If the value is greater than or equal to 10% of the maximum value of $g_{*}$, it is set to 1; otherwise, it is set to 0.

(14) $$g_{t}=Gini\left\{Y_{t}\right\}$$

(15) $$G_{t}=\left\{\begin{array}{c} 1,g_{t}>=\frac{1}{10}max\;g_{*}\\ 0,g_{t}<\frac{1}{10}max\;g_{*} \end{array}\right.$$

Nullity is defined as 1 minus the mean of $G_{t}$ calculated in Equation (15).

(16) $$NULL=1-\frac{1}{T}\sum_{t-1}^{T}G_{t}\;$$

**Figure 15:**
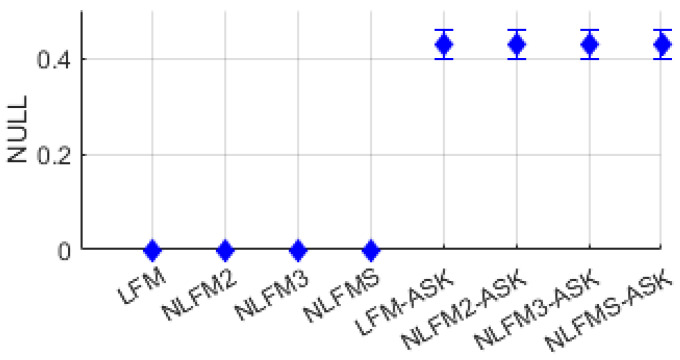
Distribution of nullity for FM, FM-ASK.

The distribution of nullity for FM single-modulation signals and FM-ASK composite-modulation signals is shown in Figure 15. It can be observed that FM single-modulation signals have a value of 0, whereas other signals have values of 0.4 or higher.

#### 2.2.9. Non-Linearity

We calculated non-linearity to estimate whether the instantaneous frequency changes linearly or non-linearly over time. First, to detect the points where the estimated instantaneous frequency changes, we identify the zero-crossing points *p* of the second derivative of $I_{t}$.

(17) $$\begin{array}{c} I_{i}^{\prime }=sign\left(I_{i+1}-I_{i}\right),i=1,\cdots ,T-1\\ I_{i}^{\prime \prime }=I_{i+1}^{\prime }-I_{i}^{\prime },i=1,\cdots ,T-2\\ p=min(\underset{i}{\arg}\;I_{i}^{\prime \prime }<0)+1 \end{array}$$

Based on the boundary points *p*, we divide $I_{t}$ into increasing frequency segments *u* and decreasing frequency segments *d*.

(18) $$\begin{array}{c} u=\left[u_{1},u_{2},\cdots ,u_{p-1}\right]=\left[I_{1},I_{2},\cdots ,I_{p-1}\right]\\ d=\left[d_{p+1},d_{p+2},\cdots ,d_{T}\right]=\left[I_{p+1},I_{p+2},\cdots ,I_{T}\right] \end{array}$$

To determine whether the elements of the vectors *u* or *d* increase or decrease linearly, we calculate the error with respect to the linear regression line of each vector *u* or *d*. The slope *a* and y-intercept *b* of the linear regression line can be obtained using the least squares method. The linear regression line vector *s* can be expressed as shown in Equation (19).

(19) $$s=\left[s_{1},s_{2},\cdots ,s_{N}\right],\;s_{n}=an+b$$

We defined nonlinearity as the mean of the error ϵ between *s* and *u*, *d*.

(20) $$\begin{array}{c} \epsilon_{u}=\frac{1}{p}\sum_{n=1}^{p-1}\left|s_{n}-u_{n}\right|\\ \epsilon_{d}=\frac{1}{T-p}\sum_{m=1}^{T-p+1}\left|s_{m}-d_{m}\right| \end{array}$$

(21) $$NL=\frac{1}{2}\left(\epsilon_{u}+\epsilon_{d}\right)$$

**Figure 16:**
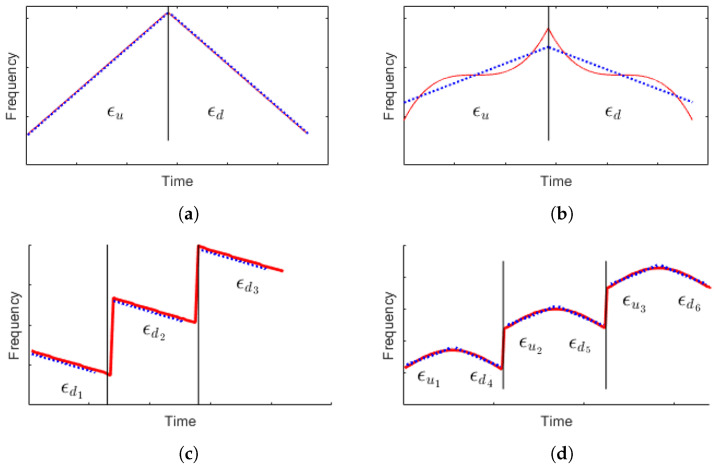
Examples of instantaneous frequency and linear regression lines for nonlinearity (**a**) LFM modulation, (**b**) NLFM3 modulation, (**c**) FSK3-LFM modulation, (**d**) FSK3-NLFMS modulation.

**Figure 17:**
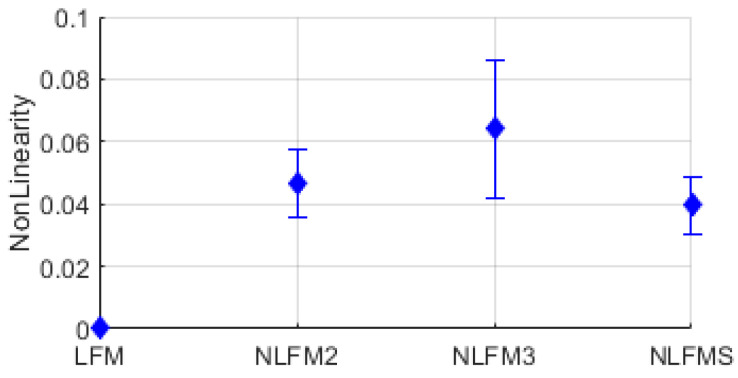
Distribution of nonlinearity for LFM, NLFM2, NLFM3, NLFMS.

Figure 16 shows examples of instantaneous frequency and linear regression lines for calculating nonlinearity for LFM, NLFM3, FSK3-LFM, and FSK3-NLFMS modulation signals. The blue dotted line and the red solid line represent the linear regression line and the instantaneous frequency, respectively. The distribution of nonlinearity for LFM, NLFM2, NLFM3, and NLFMS modulation signals is shown in Figure 17.

The 11 features described above have been summarized in Table 1.

### 2.3. Classifier Design

In this paper, we propose a classifier that combines hierarchical classification with the maximum likelihood estimation method to classify various modulation signals. We performed sequential classification of unidentified signals from modulation families to individual modulation signals based on the hierarchical classification. At each node of the hierarchical classification, we used the MLE method, which selects the parameter that maximizes the likelihood of observing the given data, instead of traditional methods based on thresholds. We modeled the Gaussian probability density function of the feature parameters and denoted the likelihood as L(f;k). In this case, the maximum likelihood estimation can be expressed as follows:

(22) $$\hat{k}=arg\max_{k}L\left(f;k\right)$$

Here, *f* is a vector composed of feature parameters, and $\hat{k}$ is the estimated value of the parameter *k* that maximizes the function. *k* consists of the means and standard deviations of the features.

We performed the following process to estimate the modulation scheme.

1. Level 0: Distinction between modulated and UnMod signals;
2. Level 1: Distinction between PSK, FSK, FM, and ASK;
3. Level 2-1: Distinction between PSK and FSK, FM;
4. Level 2-2: Distinction between LFM-ASK and NLFM-ASKl
5. Level 3: Distinction between FSK and FM;
6. Level 4-1: Distinction between FSK, FSK-FM and FSK-PSK;
7. Level 4-2: Distinction between FM-PSK and FM;
8. Level 5-1: Distinction between FSK and FSK-FM;
9. Level 5-2: Distinction between 2FSK-PSK, 3FSK-PSK, 8FSK-PSK and FSKC-PSK;
10. Level 5-3: Distinction between LFM-PSK, NLFM2-PSK, NLFM3-PSK and NLFMS-PSK;
11. Level 5-4: Distinction between LFM, NLFM2, NLFM3 and NLFMS;
12. Level 6-1: Distinction between M-ary FSK and FSKC;
13. Level 6-2: Distinction between FSK-LFM and FSK-NLFM;
14. Level 7: Distinction between 2FSK, 3FSK and 8FSK.

**Figure 18:**
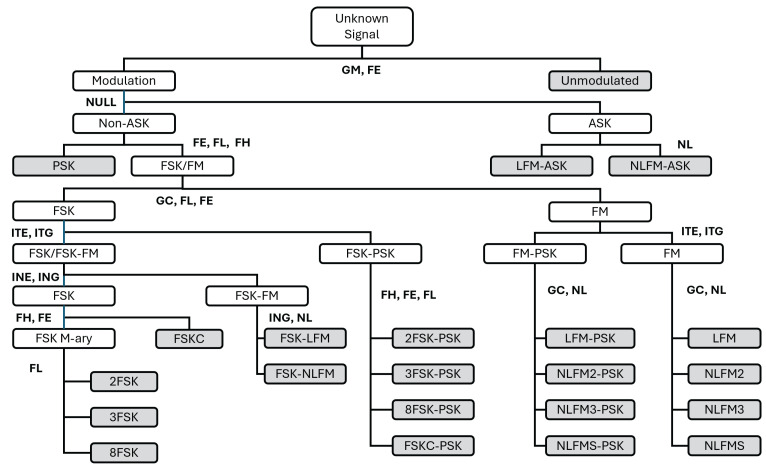
Hierarchical classification for radar modulation recognition.

The hierarchical classification for estimating the modulation signals and the features used at each node of the tree structure are shown in Figure 18. In the figure, the shaded boxes represent the leaf nodes, indicating the final classified signal class.

## 3. Experimental Result

In this study, we generated 19 types of single-modulation signals, including UnMod signals, 4 types of FM signals, 4 types of FSK modulation signals, and 10 types of PSK modulation signals. Additionally, we generated 28 types of composite-modulation signals, including 4 types of FSK-PSK modulation signals, 16 types of FSK-FM signals, 4 types of FM-ASK signals, and 4 types of FM-PSK signals. The detailed parameters for both single and composite signals are described in Table 2 and Table 3.

In Table 2 and Table 3, $f_{0}$ denotes the frequency offset, $T_{p}$ denotes the pulse width, Δf denotes the frequency gap, $T_{s}$ denotes the symbol width, ΔF denotes the bandwidth, $\gamma_{up}$ denotes the ratio of chirp-up within the pulse, *N* denotes the code length, *M* denotes the frequency step, *n* denotes the number of phase states, and *s* denotes the number of segments.

In the experiments, the SNR was set from −12 dB to 20 dB in 2 dB intervals. For each SNR, 1000 samples of each signal were generated, with 500 randomly selected samples used for training and the remaining 500 samples used for testing. To validate the performance of the proposed algorithm, cross-validation was performed 100 times, and the average accuracy was calculated.

**Figure 19:**
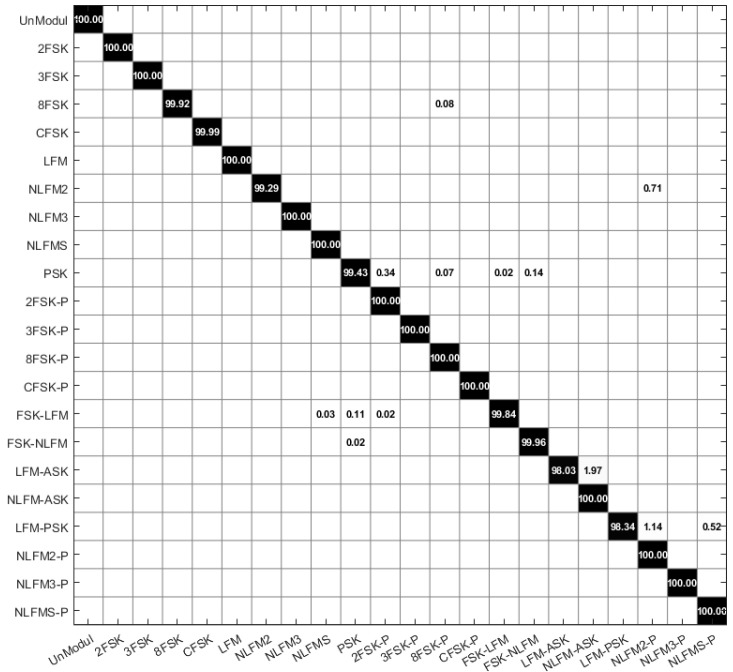
Confusion matrix for proposed method (SNR: 20 dB).

**Figure 20:**
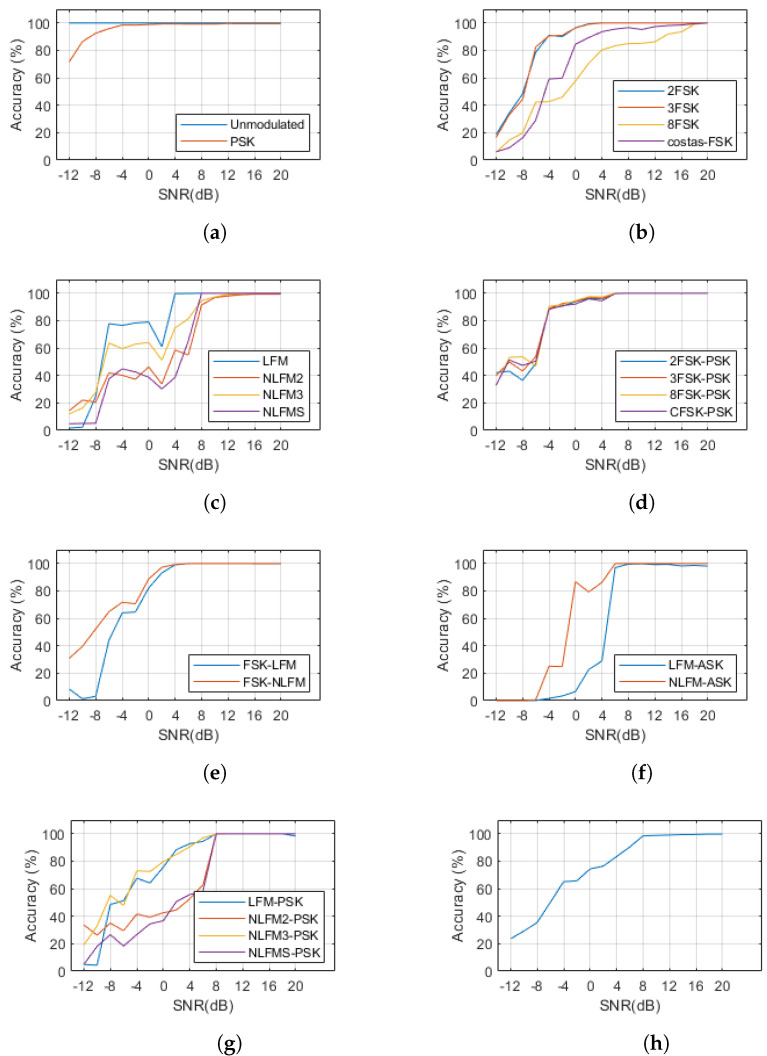
Average accuracy for each SNR of each waveform. (**a**) Unmodulated and PSK, (**b**) FSK, (**c**) FM, (**d**) FSK-PSK, (**e**) FSK-FM, (**f**) FM-ASK, (**g**) FM-PSK, (**h**) overall.

Figure 19 shows the modulation signal recognition performance in an SNR 20 dB environment as a confusion matrix. The average recognition rate is 99.76%. Figure 20 shows the change in recognition rate according to SNR. For UnMod signals, the recognition rate is 100% at an SNR of −12 to 20 dB, while, for PSK signals, the recognition rate starts to decrease below −4 dB, as shown in Figure 20a. Among the FSK single signals, it was observed that the 8FSK signal is the most affected by SNR, as shown in Figure 20b. In the case of 8FSK, the phase duration is short, making it more susceptible to noise, which likely results in an inability to accurately extract the phase duration. For FSK single signals, excluding 8FSK, it was observed that the performance significantly drops below an SNR of 4 dB. The performance for FM single signals can be seen in Figure 20c. For LFM signals, the performance is maintained up to an SNR of 4 dB, while, for NLFM signals, it is maintained up to an SNR of 8 dB. For FSK-PSK composite-modulation signals, it can be seen in Figure 20d that the performance degrades in environments with an SNR below 6 dB. Figure 20e shows that the performance for FSK-FM signals is maintained up to an SNR of 4 dB. Figure 20f shows that the performance of LFM-ASK degrades at SNRs below 6 dB, while Figure 20g shows that the performance of LFM-PSK composite-modulation signals degrades at SNRs below 8 dB. As shown in Figure 20h, the overall average performance is maintained at 98.45% up to an SNR of 8 dB, below which it drops sharply. The results of this study and related research are summarized in Table 4.

## 4. Conclusions

In this paper, we proposed a radar signal recognition method based on hierarchical classification and maximum likelihood estimation. We used STFT to obtain time–frequency images of the signals and extracted 11 feature parameters for each signal. We classified the signals using maximum likelihood estimation at each node of the hierarchical classification. The experimental results showed a performance of 98.45% in an 8 dB SNR environment.

The main contribution of this study is the ability to efficiently classify various modulation signals from broad categories to more specific ones through hierarchical classification. We effectively classified signals using maximum likelihood estimation based on a Gaussian probability model. Specifically, while related studies classified between 4 and 23 modulation signals, our study successfully classified 47 types of signals into 22 classes.

Our current research results indicate insufficient performance in low-SNR environments, and we are unable to classify PSK and LFM series signals in detail. In future research, we aim to improve not only performance in low-SNR environments but also the classification of individual modulation signals by applying noise-robust time–frequency analysis methods and developing new features.

## Figures and Tables

**Table 1:** Summary of features.

	Description
GM	Maximum power of the spectrum. Useful for distinguishing between Mod and UnMod
GC	Indicates how evenly the frequencies are distributed. Useful for distinguishing between FSK and FM, as well as between LFM, NLFM2, NLFM3, and NLFMS.
FL	Indicates the number of frequency shifts. Used to distinguish M-ary FSK signals
FE	Quantifies the uncertainty of the frequency. Used to distinguish between PSK and FSK, FM signals, as well as between FSK and FM signals.
FH	Quantifies the random change in frequency using Gini’s coefficient. Used to distinguish between PSK and FSK, FM signals
NL	Quantifies whether the instantaneous frequency increases or decreases linearly over time. Used to distinguish between LFM and NLFM signals
ITE	Quantifies, using information entropy, how evenly the lengths of the intervals where the phase does not change within a pulse are distributed. Useful for distinguishing whether there is hybrid modulation with a PSK signal or not.
ITG	Quantifies, using Gini’s coefficient (GC), how evenly the lengths of the intervals where the phase does not change within a pulse are distributed. Useful for distinguishing whether there is hybrid modulation with a PSK signal or not.
INE	Quantifies, using information entropy, whether the frequency increases or decreases within a symbol. Useful for distinguishing whether there is hybrid modulation with an FM signal or not.
ING	Quantifies, using Gini’s coefficient (GC), whether the frequency increases or decreases within a symbol. Useful for distinguishing whether there is hybrid modulation with an FM signal or not.
Nullity	Proportion of intervals where no frequency is present within the signal. Useful for distinguishing whether there is hybrid modulation with an ASK signal or not.

**Table 2:** Parameter of single modulation.

Modulation	Parameter	Value
Unmodulated	$f_{0}$	20
	$T_{p}$	10,50,100
2FSK 3FSK	$f_{c}$	20
	Δf	5, 10
	$T_{s}$	2, 5
	$T_{p}$	50, 100, 200
8FSK FSK Costas	$f_{c}$	20
	Δf	1, 2, 5
	$T_{s}$	2, 5
	$T_{p}$	50, 100, 200
LFM	$f_{c}$	20
	ΔF	20, 30, 40
	$T_{p}$	50, 100, 200
	$\gamma_{up}$	0, 0.5, 1.0
NLFM2 NLFM3 NLFMS	$f_{c}$	20
	ΔF	20, 30, 40
	$T_{p}$	50, 100, 200
PSK-Barker code	$f_{c}$	20
	*N*	7,11,13
	$T_{s}$	0.5, 1, 2, 5
	$T_{p}$	$N\times T_{s}$
PSK-PN code	$f_{c}$	20
	*N*	31, 63, 127
	$T_{s}$	0.5, 1, 2, 5
	$T_{p}$	$N\times T_{s}$
PSK-Taylor code	$f_{c}$	10
	*N*	13, 28
	$T_{s}$	0.5, 1, 2, 5
	$T_{p}$	$N\times T_{s}$
PSK-Frank code PSK P1, P2 code	$f_{c}$	20
	*M*	4, 8, 16
	$T_{s}$	0.5, 1, 2, 5
	$T_{p}$	$M^{2}\times T_{s}$
PSK P3, P4 code	$f_{c}$	20
	*N*	16, 64, 256
	$T_{s}$	0.5, 1, 2, 5
	$T_{p}$	$N\times T_{s}$
PSK T1, T2 code	$f_{c}$	20
	*n*	2, 4, 8
	*s*	4, 8, 16
	$T_{s}$	0.5, 1, 2, 5
	$T_{p}$	64, 128, 256

**Table 3:** Parameter of hybrid modulation.

Internal External	Parameter $\begin{array}{c} \left(\begin{array}{c} \mathrm{Internal}\\ \mathrm{External} \end{array}\right) \end{array}$	Value
PSK-Barker 2FSK or 3FSK	*N*	7, 11, 13
	$f_{c}$	20
	Δf	5, 10
	$T_{s}$	N,2N,5N
	$T_{p}$	$\left\lfloor \left(50,100,200\right)/T_{s}\right\rfloor /T_{s}$
PSK-Barker 8FSK	*N*	7, 11, 13
	$f_{c}$	20
	Δf	25
	$T_{s}$	N,2N
	$T_{p}$	$\left\lfloor \left(100,200\right)/T_{s}\right\rfloor /T_{s}$
PSK-Barker FSK Costas	*N*	7, 11, 13
	$f_{c}$	20
	Δf	5
	$T_{s}$	N,2N
	$T_{p}$	$\left\lfloor \left(50,100,200\right)/T_{s}\right\rfloor /T_{s}$
LFN, NLFM 2FSK or 3FSK	ΔF	Δf/2, Δf/4
	$\gamma_{up}$	0, 0.5, 1
	$f_{c}$	20
	Δf	5,10
	$T_{s}$	5, 10
	$T_{p}$	50, 100, 200
LFN, NLFM 8FSK	ΔF	Δf/2, Δf/4
	$\gamma_{up}$	0, 0.5, 1
	$f_{c}$	20
	Δf	25
	$T_{s}$	5, 10
	$T_{p}$	50, 100, 200
LFN, NLFM Costas FSK	ΔF	Δf/2, Δf/4
	$\gamma_{up}$	0, 0.5, 1
	$f_{c}$	20
	Δf	5
	$T_{s}$	5, 10
	$T_{p}$	50, 100, 200
ASK LFN, NLFM	symbol length	2, 5
	$f_{c}$	20
	ΔF	20, 30, 40
	$T_{p}$	50, 100, 200
	$\gamma_{up}$	0, 0.5, 1
PSK-Barker LFN, NLFM	code length	7, 11, 13
	$T_{s}$	2, 5
	$f_{c}$	20
	ΔF	20, 30, 40
	$T_{p}$	50, 100, 200
	$\gamma_{up}$	0, 0.5, 1

**Table 4:** Comparison between proposed method and related studies.

Authors	Kinds of LPI Radar Waveforms	Method	Noise Level	Average Accuracy
Wan, T. et al. [7]	18	kNN + SVM	0 dB	97.40%
Kishore, T.R. et al. [8]	9	Binary tree	−10 dB	90%
Ma, X. et al. [9]	5	Binary tree + kNN	−5 dB	90%
Chen, K. et al. [10]	8	IPCA + SVM	−6 dB	97.37%
Chen, K. et al. [12]	8	Deep residual learning	−8 dB	94.1%
Si, W. et al. [13]	4	DCNN	−8 dB	96.17%
Qu, Z. et al [14]	8	CNN + DQN	−6 dB	94.83%
Akyon, F.C. et al. [15]	23	FF-CNN	5 dB	99.85%
Bhatti, S.G. et al. [16]	6	BiLSTM	−2 dB	90%
Chen, K. et al. [17]	10	BLCDAE + X-net	−8 dB	96%
Proposed	47	Hierarchical classification + MLE	8 dB	98.45%

## Data Availability

Data are contained within the article.

## Abbreviations

The following abbreviations are used in this manuscript:

ASK	Amplitude Shift Keying
AWGN	Additive White Gaussian Noise
CNN	Convolution Neural Network
CWD	Choi–Williams Distribution
DCNN	Deep Convolutional Neural Network
DQN	Deep Q-Network
FE	Frequency Entropy
FF-CNN	deep Fused Fully Convolutional Neural Network
FH	Frequency Hopping
FL	Frequency Level
FM	Frequency Modulation
FSK	Frequency Shift Keying
GC	Gini’s Coefficient
GM	Gamma Max
INE	Intra-Pulse Entropy
ING	Intra-Pulse Gini’s coefficient
ITE	Inter-Pulse Entropy
ITG	Inter-Pulse Gini’s coefficient
kNN	k-Nearest Neighbor
LFM	Linear FM
MLE	Maximum Likelihood Estimation
NLFM	Non-Linear FM
PSK	Phase Shift Keying
STFT	Short-Time Fourier Transform
SVM	Support Vector Machine
UnMod	UnModulated signal
WVD	Wigner–Ville Distribution
